# RIG-I acts as a tumor suppressor in melanoma via regulating the activation of the MKK/p38MAPK signaling pathway

**DOI:** 10.1007/s13577-022-00698-1

**Published:** 2022-04-13

**Authors:** Rui Guo, Shun-Yuan Lu, Jin-Xia Ma, Qian-Lan Wang, Lu Zhang, Ling-Yun Tang, Yan Shen, Chun-Ling Shen, Jin-Jin Wang, Li-Ming Lu, Zhu-Gang Wang, Hong-Xin Zhang

**Affiliations:** 1 Research Center for Experimental Medicine, State Key Laboratory of Medical Genomics, Shanghai Ruijin Hospital, Shanghai Jiao Tong University School of Medicine, Shanghai, 200025 China; 2 Shanghai Model Organisms Center, Shanghai, 201321 China; 3 Shanghai Institute of Immunology, Shanghai Jiao Tong University School of Medicine, Shanghai, 200025 China

**Keywords:** Retinoic acid-inducible gene I, Melanoma, Proliferation, Apoptosis, p38 MAPK

## Abstract

Studies have indicated that RIG-I may act as a tumor suppressor and participate in the tumorigenesis of some malignant diseases. However, RIG-I induces distinct cellular responses via different downstream signaling pathways depending on the cell type. To investigate the biological function and underlying molecular mechanism of RIG-I in the tumorigenesis of melanoma, we constructed *RIG-I* knockout, *RIG-I*-overexpressing B16-F10 and *RIG-I* knockdown A375 melanoma cell lines, and analyzed the RIG-I-mediated change in the biological behavior of tumor cells in spontaneous and poly (I:C)-induced RIG-I activation. Cell proliferation, cell cycling, apoptosis and migration were detected by CCK-8 assay, BrdU incorporation assay, Annexin V–PI staining assay and Transwell assay, respectively. In vivo tumorigenicity was evaluated by tumor xenograft growth in nude mice and subsequently by Ki67 staining and TUNEL assays. Furthermore, Western blotting was utilized to explore the underlying mechanism of RIG-I in melanoma cells. Our data showed that RIG-I promotes apoptosis and inhibits proliferation by G1 phase cell cycle arrest in the melanoma cell lines. Mechanistically, RIG-I induced the phosphorylation of p38 MAPK and MAPK kinases MKK3 and MKK4. In conclusion, the current study demonstrated that RIG-I suppressed the development of melanoma by regulating the activity of the MKK/p38 MAPK signaling pathway, which is relevant to research on novel therapeutic targets for this malignant disease.

**Supplementary Information:**

The online version contains supplementary material available at 10.1007/s13577-022-00698-1.

## Introduction

Melanoma is one of the most malignant and aggressive types of skin cancer, accounting for more than 80% of skin cancer-associated deaths worldwide [1]. In the past several decades, the annual incidence and range of onset areas of melanoma have increased rapidly [2]. Although many therapeutic methods, including surgical resection, combined chemotherapy, photodynamic therapy, radiotherapy and molecular-targeted therapy, have been used extensively for the treatment of melanoma, the overall 5 year survival rate for patients remains poor [3, 4]. Therefore, there is an urgent need to investigate melanoma biology and identify novel risk factors for the development of melanoma, which will be helpful in finding effective methods for the prevention, diagnosis and early treatment of this disease. At present, a number of factors have been demonstrated to be associated with the development of melanoma, including phenotypic, genetic and environmental risk factors [5, 6]. In the last several decades, a number of genes related to the pathogenesis of melanoma have been identified by genetic studies [7–9].

Retinoic acid-inducible gene I (RIG-I), also known as DDX58, is the charter member of the RLR (RIG-I-like receptors) family, and is an important cytosolic virus recognition molecule involved in innate antiviral immune responses [10]. RIG-I is a kind of DExD/H box-containing RNA helicase and consists of two amino (N)-terminal caspase activation and recruitment domains (CARDs) in tandem, a central DExH/C box ATPase domain and a carboxy-terminal regulatory/repressor domain (RD) [11]. In the absence of ligand RNA, RIG-I adopts an autoinhibitory conformation. After binding to appropriate RNA, it undergoes conformational changes that expose CARDs and then binds and activates the adaptor protein MAVS (mitochondrial antiviral signaling protein) via homotypic CARD–CARD interactions [12–14]. MAVS subsequently acts as a scaffold by recruiting and activating downstream signaling molecules, including TBK1/IKK-i and IKKα/IKKβ complexes, which further facilitates the activation of the transcription factors IRF3/IRF7 and NF-κB, respectively. Activated IRF3 and NF-κB then induce the expression of type I interferons and some other related genes that eventually provide an antiviral state for host cells [10, 15]. Intriguingly, accumulating evidence indicates that RIG-I is also involved in a series of cellular and physiological processes, such as inflammation and inflammatory diseases, cell proliferation, apoptosis, senescence and even carcinogenesis [16, 17]. Our previous work reported that RIG-I-deficient mice developed a colitis-like phenotype and were prone to colitis-associated colorectal cancer [18, 19]. Other studies also indicate that RIG-I may function as a tumor suppressor and that RIG-I may involve different signaling pathways in a cell-specific manner [20, 21].

Several studies have revealed that RIG-I participates in the progression of interferon-α-induced apoptosis in melanoma tumor-repopulating cells [22] and is involved in the apoptosis of melanoma cells in response to its agonist poly (I:C) [23]. However, the precise roles of RIG-I in pathogenesis and treatment have not yet been fully elucidated. To further clarify the biological function of RIG-I in the pathology of melanoma, we analyzed the effects of RIG-I on tumor behavior with and without poly (I:C) using *RIG-I*-knockout (KO), *RIG-I*-overexpressing murine B16-F10 and human A375 melanoma cell lines. Our data indicated that RIG-I participates in proliferation and apoptosis via the MKK-p38 MAPK signaling cascade. These findings reveal an important novel role of RIG-I in the tumorigenesis of melanoma.

## Materials and methods

### Cell lines and cell culture

B16-F10 murine melanoma cells and A375 human melanoma cells were obtained from the Cell Bank of Shanghai Institute of Cell Biology (Shanghai, China). Cells were maintained in high-glucose Dulbecco’s modified Eagle’s medium (DMEM) (HyClone, USA) supplemented with 10% fetal bovine serum (FBS, Gibco, USA). Cells were cultured at 37 °C in a humidified 5% CO_2_ atmosphere.

### Knockout and overexpression of *RIG-I*

The *RIG-I*-knockout B16-F10 melanoma cell line was generated using the CRISPR–Cas9 gene editing system. Briefly, the guide-RNA for *RIG-I* was designed with the online CRISPR design tool (https://zlab.bio/guide-design-resources) to target exon 1 of *RIG-I* gene, and the two complementary oligonucleotides were as follows: sgRNA-1: 5'-CTACATGAGTTCCTGGCTCG-3' and sgRNA-2: 5'-AAACCGAGCCAGGAACTCATGTAGC-3'. The Cas9 PX459 plasmid was digested with BbsI and gel purified. The oligonucleotides were annealed and cloned into the BbsI-digested Cas9 PX459 plasmid. 4 μg of RIG-I Cas9-PX459 plasmids were transfected into B16-F10 cells using Lipofectamine 3000 Transfection Reagent (Invitrogen, USA) and the empty vector was used as the control. After 48 h, the cells were incubated in DMEM with 4 μg/mL puromycin for 7 days. Subsequently, single *RIG-I*-KO and control cell clones were identified by western blot analysis and DNA sequencing by specific primers (PCR forward primer, 5′-CCGGCTAGGCAGCTTTTTCATC-3′; PCR reverse primer, 5′-CCATCAAATCCCTATGCTGTCTACTC-3′; sequencing primer, 5′-GGAAAGTCCCCACTTCGTTCATCTC-3′). The expression vector of RIG-I has been described previously [24]. Briefly, full-length *RIG-I* cDNA was amplified by PCR using primers with Kpn I or Not I sites (5′-ACTGGTACCATGACCGCGGCGCAGCGGCA-3′ and 5′-ACTGCGGCCGCTCATACGGACATTTCTGCAG-3′). The PCR products were digested with Kpn I and Not I and cloned into the corresponding sites in the pCMV-Myc vector. The vector was confirmed by sequencing and further transfected into B16-F10 cells using Lipofectamine 3000 Transfection Reagent (Invitrogen, USA), according to the manufacturer’s instructions.

### Small interfering RNA transfection

The nontargeting control (NC) and human RIG-I specific siRNA (Si-RIG-I) was synthesized by Sango Biotech (Shanghai, China). The siRNA sequences were the following: NC (forward), 5′-UUCUCCGAACGUGUCACGUTT-3′; NC (reverse), 5′-ACGUGACACGUUCGGAGAATT-3′; Si-RIG-I (forward), 5′-CCAGAAUUAUCCCAACCGAUATT-3′; Si-RIG-I (reverse), 5′-UAUCGGUUGGGAUAAUUCUGGTT-3′. A375 cells in the logarithmic growth phase were carefully selected and transfected with 100 nM indicated siRNAs after they reached 70% confluency by using Lipofectamine RNAiMAX (Invitrogen, USA), according to the manufacturer’s protocol. Western blotting analysis was used to identify the effect of *RIG-I* knockdown.

### Poly (I:C) treatment

The cells were cultured in fresh complete medium and treated with poly (I:C) (10 μg/ml) for 24 h using Lipofectamine 3000 Transfection Reagent (Invitrogen, USA), according to the manufacturer’s instructions.

### Western blotting

Cells were collected and lysed using RIPA buffer (Beyotime, China) with fresh protease inhibitor cocktail and phosphatase inhibitor (Roche, Switzerland), and the cell lysates were collected and centrifuged at 12,000 rpm and 4 °C for 10 min. The supernatants were collected carefully, and the concentration was quantified by a BCA Protein Assay Kit (Thermo Fisher, USA). Each sample contained an equal concentration of 30 µg of protein. The samples were heated at 100 °C for 5 min, and 20 µL of each sample was loaded onto an 8% sodium dodecyl sulfate (SDS)-polyacrylamide gel and electrophoresed. Isolated proteins were transferred onto a nitrocellulose filter (NC) membrane and then blocked with 5% nonfat skim milk in PBS and shaken for 1 h. The membranes were incubated with primary antibodies against RIG-I (1:1000, D14G6, Cell Signaling, USA), P-MKK3 (1:1000, 12,280, Cell Signaling, USA), MKK3 (1:1000, 8535, Cell Signaling, USA), P-MKK4 (1:1000, 4514, Cell Signaling, USA), MKK4 (1:1000, 9152, Cell Signaling, USA), P-p38 (1:1000, 9211, Cell Signaling, USA), p38 (1:1000, 9212, Cell Signaling, USA), P-AKT (1:2000, 4060, Cell Signaling, USA), AKT (1:2000, 4691, Cell Signaling, USA), P-ERK (1:1000, 4370, Cell Signaling, USA), ERK (1:1000, 9102, Cell Signaling, USA), Cyclin D1 (1:1000, 2978, Cell Signaling, USA), and GAPDH (1:2000, D110016, BBI, China) overnight at 4 °C. Then, the membranes were washed three times with PBST for 5 min, incubated with IRDyeCW800-conjugated anti-rabbit immunoglobulin (LI-COR) for 1 h at room temperature and scanned with the LI-COR Odyssey imaging system (LI-COR, USA).

### CCK8 cell viability assay

A total of 6000 B16-F10 or A375 cells in 100 μL complete medium were seeded into 96-well plates. After being attached, the cells were transfeced with or without 10 μg/ml poly (I:C) at 37 °C in a humidifier 5% CO_2_ atmosphere for 24 h. According to the protocol of CCK8 solution (Dojindo, Japan), 10 μL of CCK-8 diluted in 100μL of fresh complete medium replaced the original culture medium of each group per 24 h and the cells were incubated for 1 h at 37 °C. The viable cells were detected by using absorbance at 450 nm wavelength using a microplate reader (BioTek Instruments, USA).

### BrdU incorporation and apoptosis detection

B16-F10 or A375 melanoma cells were plated into six-well plates (2 × 10^5^/per well). After 24 h, the cells were treated with or without poly (I:C) for 24 h. For apoptosis assays, cells were harvested and marked with APC-Annexin V and PI using an Annexin V APC Apoptosis Detection Kit (eBioscience, USA) according to the manufacturer’s instructions. For proliferation assays, cells were loaded with BrdU (5 µg/ml) for 1 h, stained with anti-BrdU and then marked with 7-AAD using a BrdU Cell Proliferation Kit (Biolegend, USA). The results were evaluated with FlowJo software (BD Bioscience, USA).

### Cell migration assay

Migration assays were performed using Millipore Transwell system chambers (8 μm pore size, Millipore, USA). A total of 5 × 10^4^ cells were seeded into the upper chambers of 24-well plates in 400 μl serum-free DMEM, while the lower chambers were filled with 600 μl complete medium with 3 μg/ml mitomycin. The plate was incubated at 37 °C for 48 h. Then, the cells in the upper chambers were removed with a cotton swab, and the lower chambers were fixed with 4% PFA for 10 min at room temperature, stained with 0.5% crystal violet in 10% methanol and photographed at × 200 magnification.

### In vivo experiments

Fourteen 6 week-old specific pathogen-free BALB/c female nude mice weighing 20–25 g were purchased from Phenotek Biotechnology (Shanghai) Co., Ltd. and maintained in specific pathogen-free conditions, with each mouse in an independent ventilation cage at 20–26 °C with a humidity of 40–70% and a 12/12-h light/dark cycle (lights on at 06:00 a.m.). Mats and feed were changed every 3 days. For the tumor challenge, 5 × 10^5^ *RIG-I* knockout B16-F10 melanoma cells or control B16-F10 melanoma cells in 50 µl of serum-free DMEM were injected subcutaneously into the right flank area of the nude mice. The mice were randomly divided into two groups using a computer-based random order generator. There were seven animals in each group. One group of mice was injected with control B16-F10 melanoma cells, while the other group of mice was injected with *RIG-I* knockout B16-F10 cells. For inclusion in the experimental and control groups, a mouse was randomly selected and permanently marked with a number. Measurements of tumor growth were started when the tumors grew 2 to 3 mm in diameter and were visible. Tumors were observed daily, and the size of the tumors was measured every 3–4 days. The measurement time is from 2:00 p.m. to 4:00 p.m. and the measurement sequence is in accordance with the number of mice. The mice were killed on day 20 after implantation. All mice were euthanized with CO_2_. The initial process of CO_2_ delivery to the microisolator is completed by opening the CO_2_ cylinder valve so that the mice will slowly be exposed to increasing CO_2_ levels (displacement per minute is approximately 30% of the cavity volume). After the color of the eyes disappeared and breathing stopped, the flow of carbon dioxide was maintained for 5 min to confirm death. Tumor tissues were prepared for immunostaining. All experimental manipulations were approved by the Animal Ethics Committee of Ruijin Hospital Affiliated to Shanghai Jiao Tong University School of Medicine. Our research was in compliance with the institutional guidelines for the care and use of animals.

### Immunofluorescence

The tumor tissue samples were fixed with 4% paraformaldehyde at 4 °C overnight, embedded in paraffin and cut into 3 µm-thick sections. The sections were deparaffinized, rehydrated, and subsequently subjected to antigen retrieval. The sections were blocked in blocking buffer (10% goat serum in 0.5% Triton X-100) for 30 min and then incubated with a primary antibody against Ki67 (1:1000, ab15580, Abcam, USA) overnight at 4 °C. Then, the slices were washed with 0.5% Triton X-100 three times and incubated with Alexa Flour 594 chicken anti-rabbit IgG (H + L) (1:500, A21442, Invitrogen, USA) and DAPI (1:1000, D9542, Sigma, USA) for 2 h at room temperature. Apoptotic cells in the tumor tissue sections were quantified using the in situ Cell Death Detection Kit (Roche, Switzerland). The sections were visualized by a fluorescent microscope (Nikon, Japan) using appropriate excitation and emission spectra at 400 × magnification.

### Statistical analysis

Statistical analysis was performed with GraphPad Prism (version 7.0, USA). All data are shown as the mean ± SD. To compare the counts of Ki67-positive cells and TUNEL-positive cells in mice, Student’s *t* test was used. To compare tumor volumes, CCK8 cell viability assays, BrdU assays and APC-Annexin V assays, two-way ANOVA was used. Statistical significance was defined as *P* < 0.05.

## Results

### Generation of *RIG-I*-KO cell lines with the CRISPR–Cas9 gene editing system

**Fig. 1:**
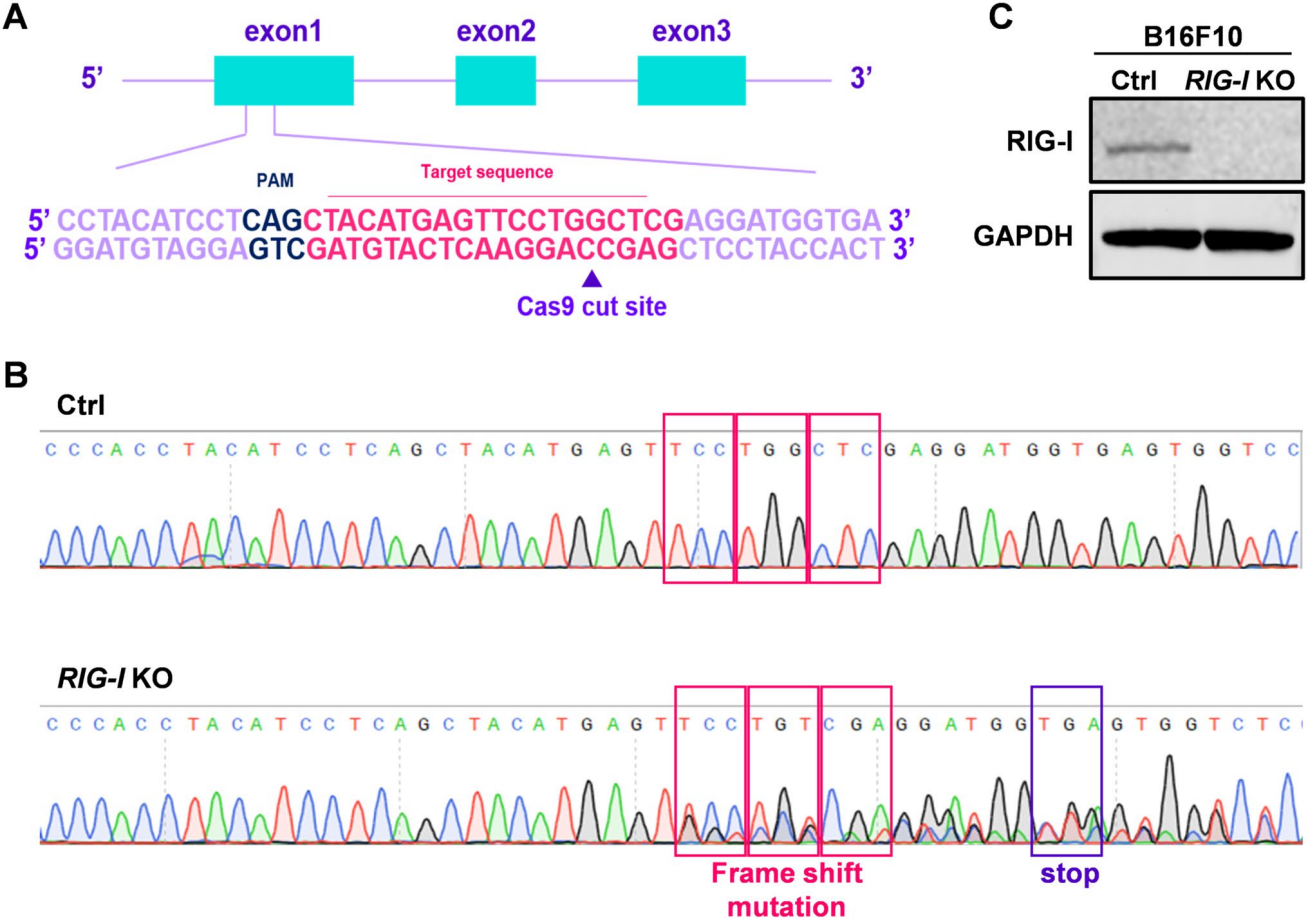
Generation and validation of *RIG-I* knockout in B16-F10 cells. **A** Schematic of the gRNA target site of *RIG-I* knockout cells. The PAM sequence is marked in dark blue. The arrow indicates the cleavage site by Cas9. **B** Two bases of nucleic acid were deleted, resulting in frameshift mutation and translation termination by a stop codon after the mutant site in *RIG-I* KO B16-F10 cells by DNA sequencing. **C** The control and *RIG-I* KO cells were analyzed by Western blotting with anti-RIG-I antibody. Anti-GAPDH antibody was used as a loading control

The CRISPR–Cas9 gene editing system was used to generate a *RIG-I*-knockout melanoma cell line. The oligonucleotides for the guide RNA were designed, synthesized and cloned into the PX459 vector (Fig. 1A). Then, the vectors were transfected into B16-F10 cells. The indel mutations in these cell lines were confirmed by DNA sequencing of the PCR products of target DNA (Fig. 1B). Finally, Western blotting with an anti-RIG-I antibody showed that the expression of RIG-I protein was abolished in the *RIG-I*-KO cell line (Fig. 1C).

### RIG-I plays a suppressive role in the proliferation of melanoma cells

**Fig. 2:**
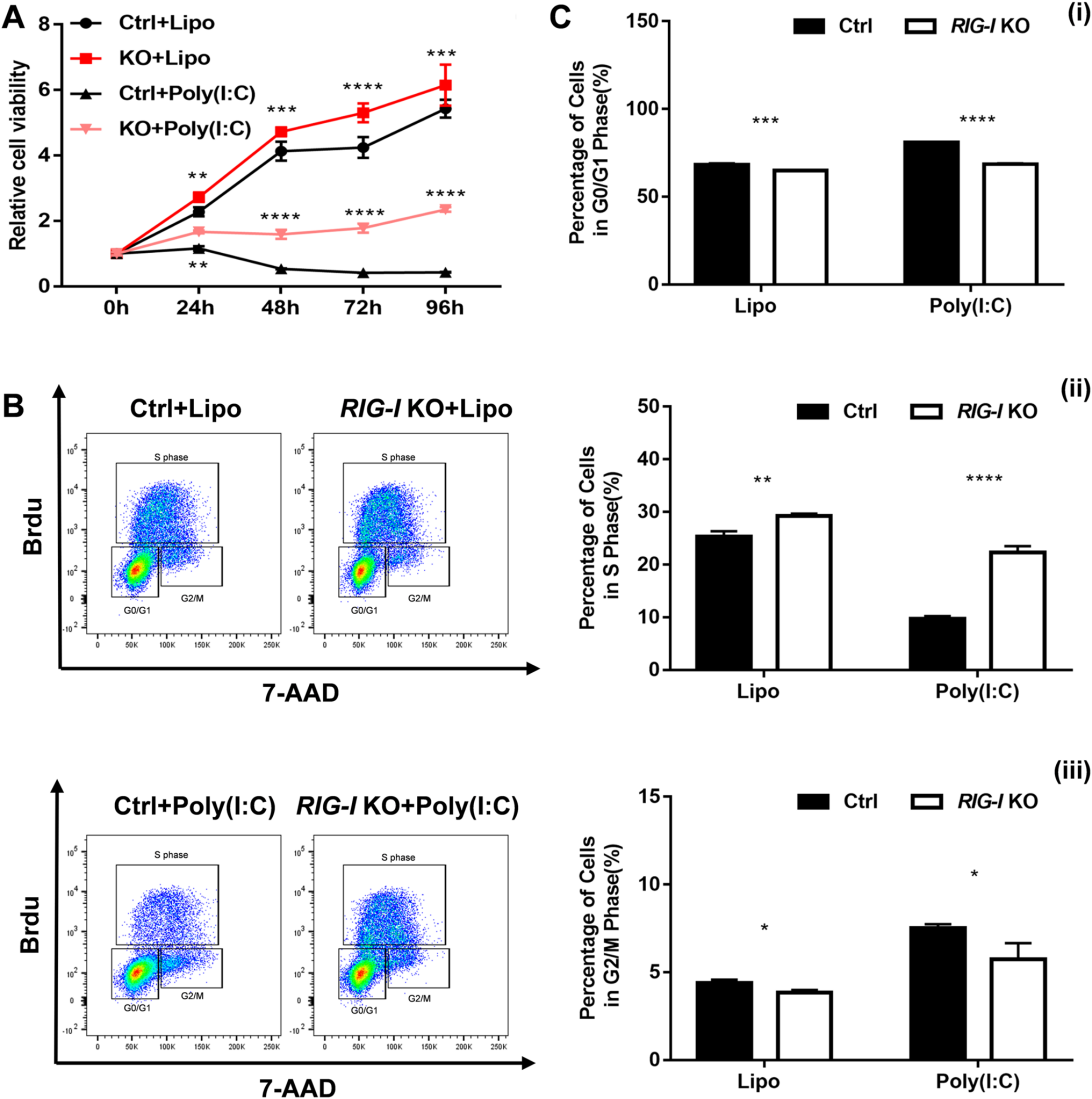
Target deletion of *RIG-I* promotes the proliferation of B16-F10 cells. **A** CCK-8 assay was performed to evaluate the cell viability in Ctrl and *RIG-I* KO B16-F10 cells with or without poly (I:C) (10 μg/ml). *n* = 4, ***p < 0.001. **B** BrdU incorporation and FACS analyses for control and *RIG-I* KO B16-F10 cells treated with poly (I:C) (10 μg/ml) or Lipofectamine 3000. **C** Percentages of different stages of the cell cycle were calculated. *n* = 3 in each group. (1) Percentage of cells in the G0/G1 phase. (2) Percentage of cells in the S phase. (3) Percentage of cells in the G2/M phase. *P < 0.05, **P < 0.01, *** *P* < 0.001, **** *P* < 0.0001. Three independent experiments were performed

**Fig. 3:**
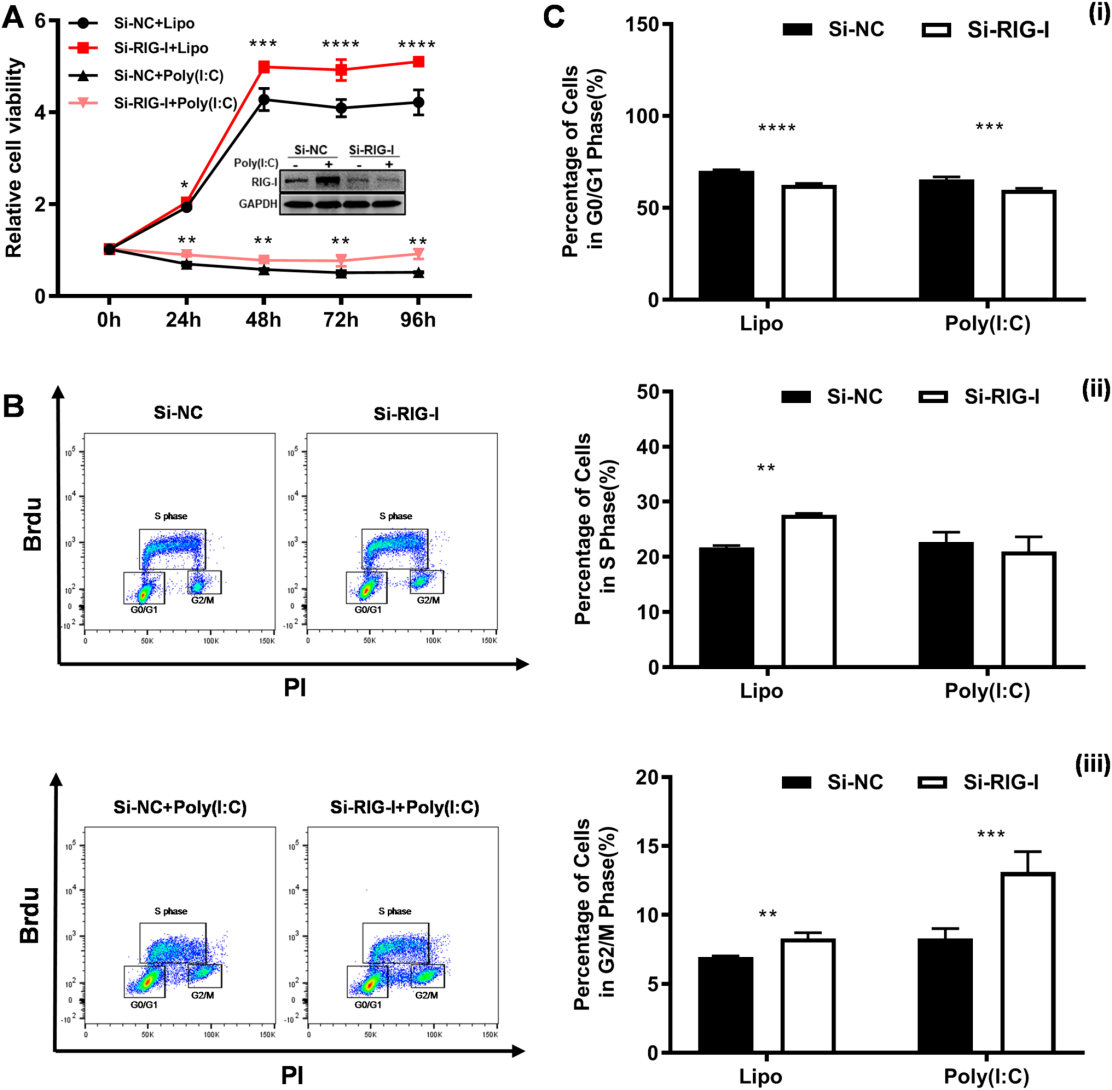
*RIG-I* knockdown promotes the proliferation of A375 cells. **A** A375 cells were transfected with Si-NC and Si-RIG-I. 24 h later, the cells were treated with or without poly (I:C) (10 μg/ml). The expression of RIG-I was determined by Western blotting and the cell viability was evaluated by CCK-8 assay. *n* = 4, ***p < 0.001. **B** BrdU incorporation and FACS analyses for Ctrl (Si-NC) and *RIG-I* knockdown (Si-RIG-I) A375 cells treated with poly (I:C) (10 μg/ml) or Lipofectamine 3000. **C** Percentages of different stages of the cell cycle were calculated. *n* = 3 in each group. (1) Percentage of cells in the G0/G1 phase. (2) Percentage of cells in the S phase. (3) Percentage of cells in the G2/M phase. **P < 0.01, *** *P* < 0.001, **** *P* < 0.0001. Three independent experiments were performed

The effect of RIG-I deficiency on the growth of B16-F10 cells was first evaluated by CCK-8 assay. As shown in Fig. 2A, the relative cell viability of B16-F10 cells was significantly increased in the *RIG-I* knockout group compared with that in the control group, both in the spontaneous and poly (I: C)-stimulated state. To further elucidate the effects of *RIG-I* knockout on the proliferation capacity of B16-F10 cells, we performed a BrdU incorporation assay and assessed the cell cycle distribution using flow cytometry analysis. The results showed that target deletion of RIG-I resulted in a decreased percentage of cells in the G0/G1 phase (spontaneous: 64.97 ± 0.15 vs. 68.17 ± 0.85%, *P* < 0.001; poly (I: C)-stimulation: 68.6 ± 0.50 vs. 80.93 ± 0.32%, *P* < 0.0001) and an increased percentage of cells in the S phase (spontaneous: 29.23 ± 0.46 vs. 25.3 ± 1.05%, *P* < 0.01; poly (I: C)-stimulation: 22.30 ± 1.22% vs. 9.72 ± 0.52%, P < 0.0001) (Fig. 2B, C). Moreover, RIG-I siRNA-transfected A375 human melanoma line showed significantly increased cell viability (Fig. 3A). The results of BrdU incorporation assay also showed that *RIG-I* knockdown resulted in a decreased percentage of cells in the G0/G1 phase, and increased percentage of cells in the G2/M and S phase (Fig. 3B, C). These data suggested that RIG-I deficiency promotes melanoma cell proliferation.

**Fig. 4:**
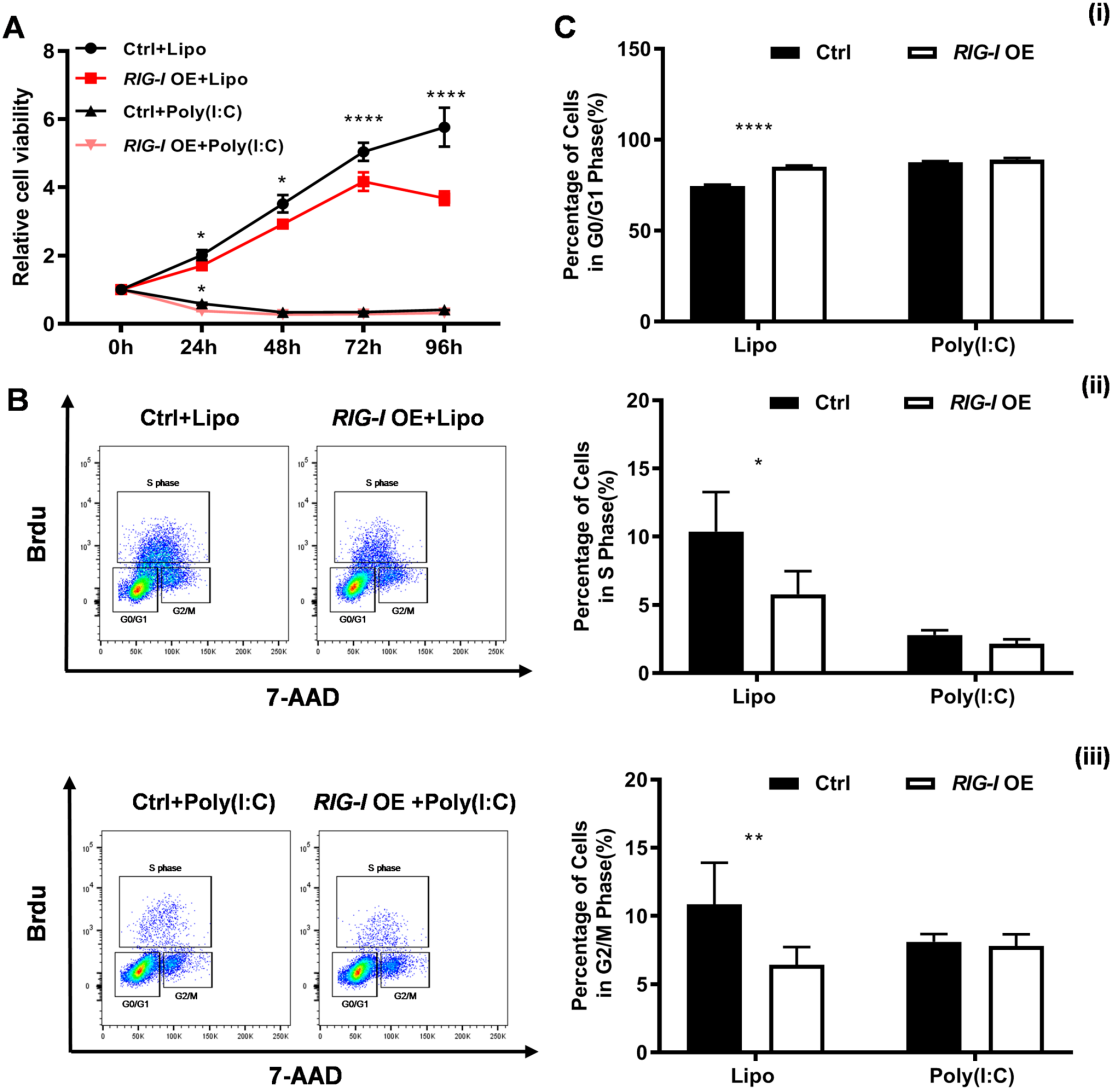
Ectopic overexpression of *RIG-I* (*RIG-I* OE) inhibits the proliferation of B16-F10 cells. **A** CCK-8 assay was performed to evaluate the viability of Ctrl and *RIG-I*-overexpressing B16-F10 cells with or without poly (I:C) (10 μg/ml). *n* = 4, ***p < 0.001. **B** BrdU incorporation and FACS analyses for control and *RIG-I* OE B16-F10 cells treated with poly (I:C) (10 μg/ml) or Lipofectamine 3000. **C** Percentages of different stages of the cell cycle were calculated. *n* = 3 in each group. (1) Percentage of cells in the G0/G1 phase. (2) Percentage of cells in the S phase. (3) Percentage of cells in the G2/M phase. *P < 0.05, **P < 0.01, **** *P* < 0.0001. Three independent experiments were performed

To further explore the role of RIG-I in the proliferation of melanoma cells, cell viability and cell cycle distribution were assessed in B16-F10 cells after transfection with *RIG-I*-expressing vector or control vector. The CCK-8 results showed that ectopic forced expression of RIG-I led to dramatic suppression of cell viability compared with control cells (Fig. 4A). As shown in Fig. 4B, C, *RIG-I* overexpression significantly increased the percentage of cells in the G0/G1 phase (85.13 ± 0.64 vs. 74.45 ± 0.85%, *P* < 0.0001) and decreased the percentage of cells in the S phase (5.46 ± 1.58 vs. 10.36 ± 2.90%, *P* < 0.05) and G2/M phase (6.42 ± 1.30% vs. 10.83 ± 3.07%, *P* < 0.01) in B16-F10 cells. Most importantly, poly (I:C)-induced RIG-I activation also suppresses the proliferation in *RIG-I*-overexpressing and control cells, but not in *RIG-I*-KO cells. These data indicated that RIG-I is able to suppress the proliferation of melanoma cells by arresting the cell cycle in the G1 phase.

### RIG-I plays a positive regulatory role in the apoptosis of melanoma cells

**Fig. 5:**
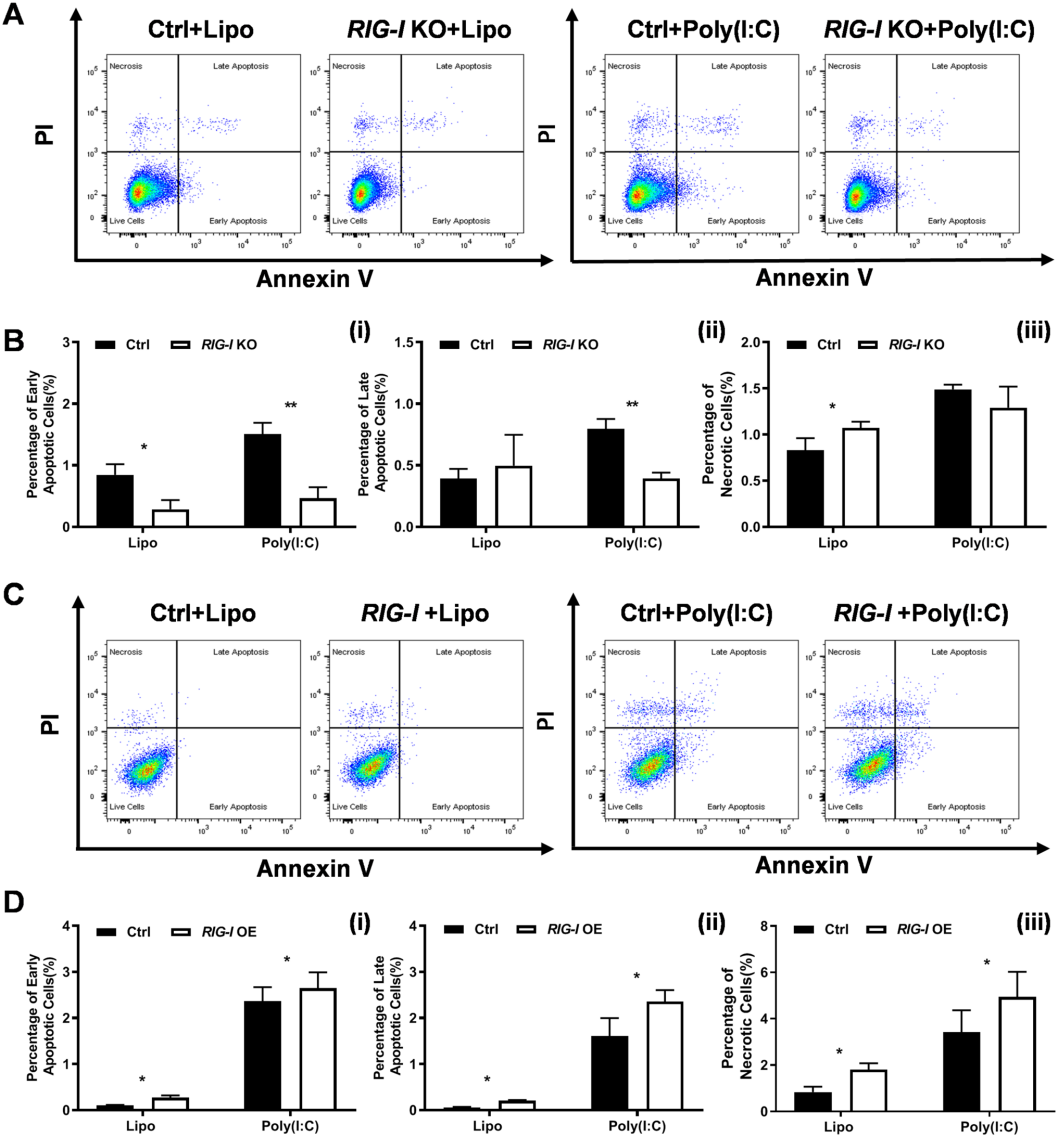
Effects of *RIG-I* KO and *RIG-I*-overexpressing (*RIG-I* OE) on the apoptosis of B16-F10 cells. **A** Flow cytometry analysis of apoptosis in Ctrl and RIG-I KO B16-F10 cells treated with poly (I:C) (10 μg/ml) or Lipofectamine 3000. **B** Percentages of different stages of apoptotic cells were calculated. (1) Early apoptosis: Annexin V^+^/PI^−^, (2) Late apoptosis: Annexin V^+^/PI^+^, (3) Necrosis: Annexin V^−^/PI^+^. *P < 0.05, **P < 0.01. **C**, **D** Flow cytometry analysis of apoptosis in Ctrl and *RIG-I*-overexpressing B16-F10 cells. *P < 0.05. Three independent experiments were performed

**Fig. 6:**
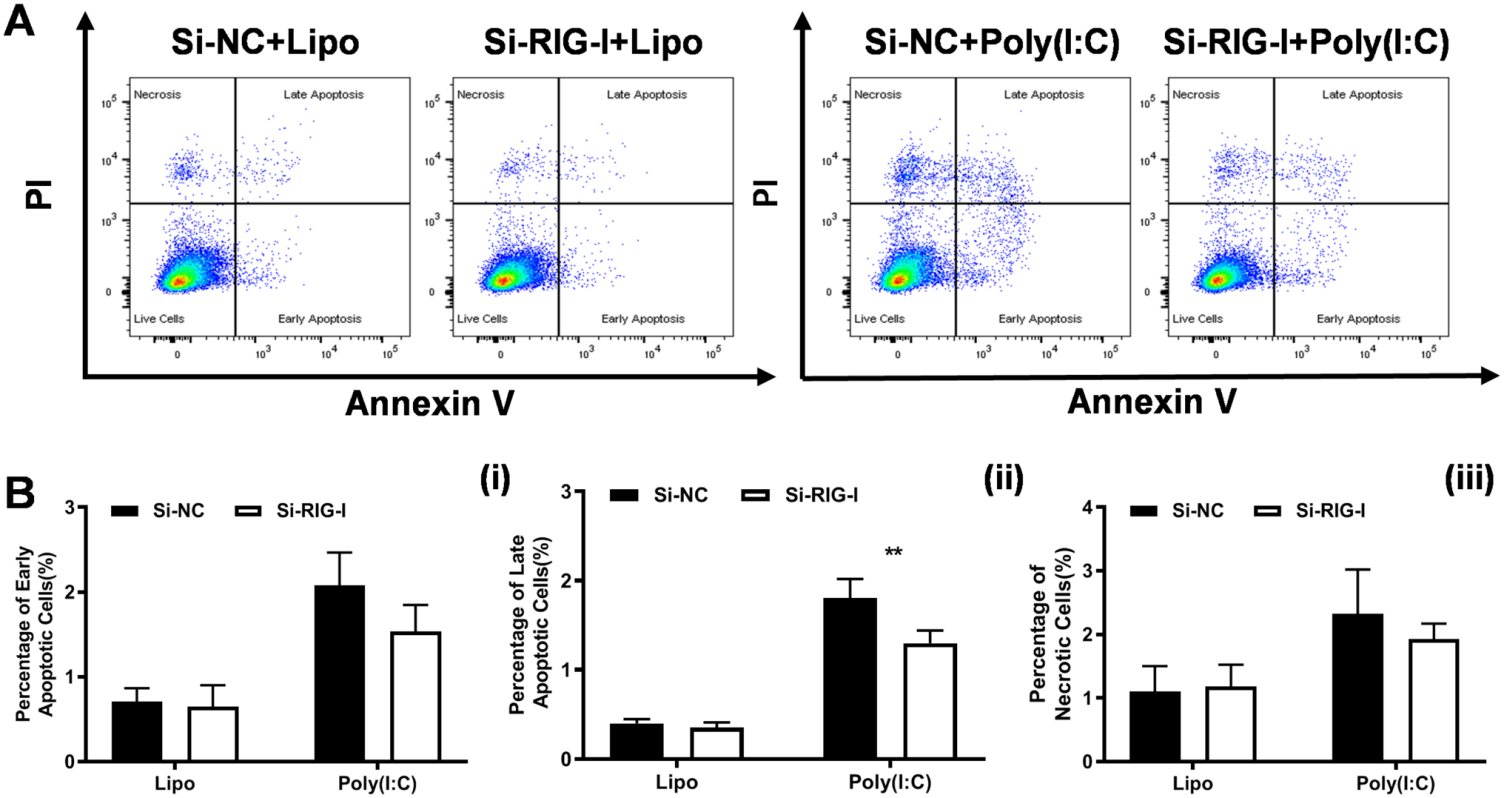
Effects of *RIG-I* knockdown on the apoptosis of A375 cells. **A** Flow cytometry analysis of apoptosis in Ctrl (Si-NC) and *RIG-I* knockdown (Si-RIG-I) A375 cells treated with poly (I:C) (10 μg/ml) or Lipofectamine 3000. **B** Percentages of different stages of apoptotic cells were calculated. (1) Early apoptosis: Annexin V^+^/PI^−^, (2) Late apoptosis: Annexin V^+^/PI^+^, (3) Necrosis: Annexin V^−^/PI^+^. **P < 0.01. Three independent experiments were performed

To clarify the effects of RIG-I on the apoptosis of melanoma cells, we performed an Annexin V/PI staining assay and flow cytometry analyses. The results showed that the percentage of early apoptotic cells (Annexin V^+^/PI^−^, 0.28 ± 0.15 vs. 0.85 ± 0.17%, *P* < 0.05) was significantly decreased in *RIG-I*-KO cells compared with control cells (Fig. 5A, B). We further observed that transfection of poly (I:C) could induce dramatic cell apoptosis. Both the poly (I:C)-induced early (Annexin V^+^/PI^−^, 0.47 ± 0.18 vs. 1.51 ± 0.19%, *P* < 0.01) and late apoptosis (Annexin V^+^/PI^+^, 0.39 ± 0.05% vs. 0.80 ± 0.08%, *P* < 0.01) were significantly inhibited in the *RIG-I* KO group. Then, the effects of *RIG-I* overexpression on cell apoptosis were studied. The data revealed that both early (0.26 ± 0.06 vs. 0.10 ± 0.01%, *P* < 0.05) and late apoptosis (0.21 ± 0.04 vs. 0.06 ± 0.01%, P < 0.05) were dramatically increased in *RIG-I*-overexpressing melanoma cells compared with control cells. As expected, ectopic forced expression of RIG-I significantly enhanced poly (I: C)-induced apoptosis (early apoptosis: from 2.37 ± 0.30 to 2.81 ± 0.17%, *P* < 0.05; late apoptosis: from 1.61 ± 0.39 to 2.07 ± 0.56%, *P* < 0.05) and necrosis (from 3.42 ± 0.95 to 4.77 ± 0.79%, *P* < 0.05) (Fig. 5C, D). Furthermore, we evaluated the role of RIG-I in the apoptosis of A375 human melanoma cell line. The results showed that *RIG-I* knockdown A375 cells also showed significantly increased poly (I: C)-induced apoptosis (Fig. 6). These results further confirmed the central role of RIG-I in poly (I: C)-induced effects on the melanoma cell line.

### RIG-I depletion does not affect the migration of melanoma cells

To explore the role of RIG-I in the metastasis of melanoma, we assessed cell migration in vitro. Although the cell–matrix interaction was dramatically increased in the *RIG-I*-KO group, the Transwell assay showed that the number of cells that migrated out in the *RIG-I*-KO group was comparable with that in the control group with or without poly (I:C) (Figure S1). These data indicated that RIG-I is not the core factor in melanoma metastasis.

### Target deletion of RIG-I facilitates the growth of tumor xenografts in vivo

**Fig. 7:**
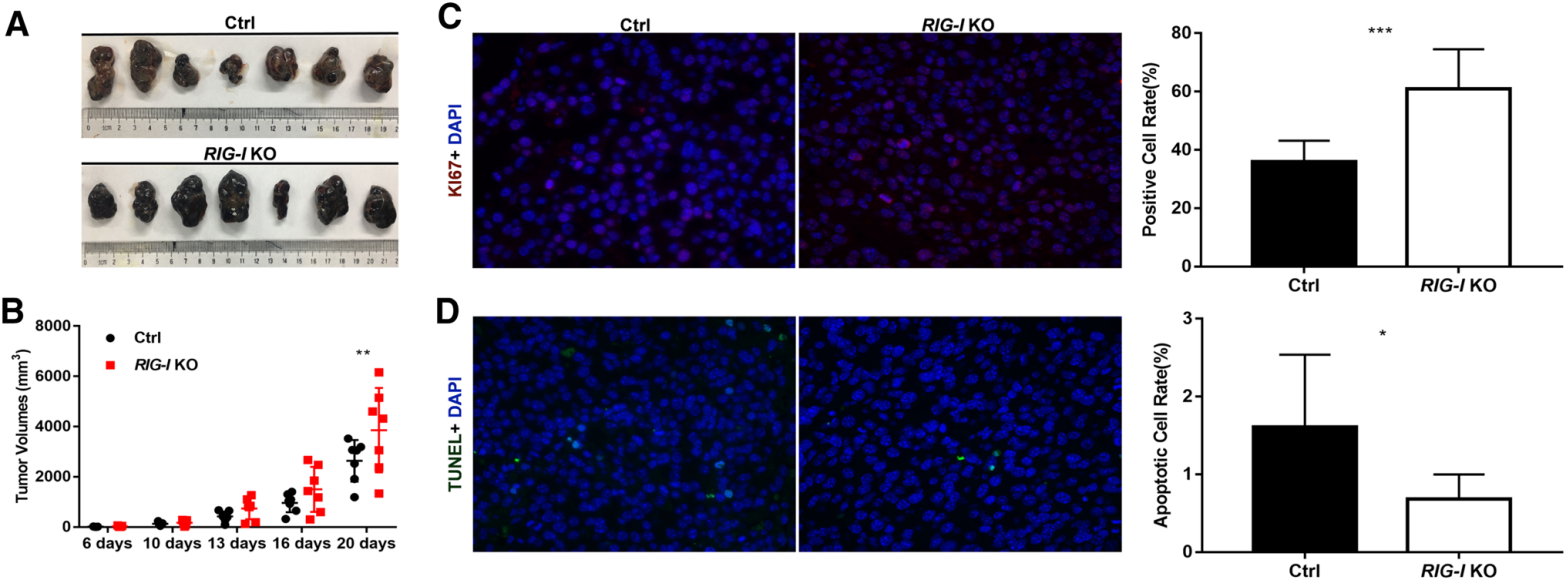
Knockout of *RIG-I* enhances the growth of B16-F10 cell xenografts in a nude mouse model. **A** Images of tumors isolated from the nude mouse model derived from *RIG-I* KO and control B16-F10 melanoma cells (*n* = 7). **B** Tumor volume was calculated by the following formula: tumor Volume (mm^3^) = (length × width^2^)/2. **P < 0.01. **C** Cell proliferation was assessed by Ki67 staining. Ki67-positive cell proportions were quantified by ImageJ and calculated as follows: positive Cell Rate (%) = Ki67^+^ cells (depicted as red fluorescence)/total number of cells per photo (represented by blue nuclei stain, DAPI). **D** TUNEL staining was utilized to measure melanoma cell apoptosis in tumors. TUNEL-positive cell proportions were quantified by ImageJ and calculated as follows: apoptotic Cell Rate (%) = TUNEL-positive cells (depicted as green fluorescence)/total number of cells per photo (represented by blue nuclei stain, DAPI). *P < 0.05, ***P < 0.001. Three independent experiments were performed

After it was determined that RIG-I promoted apoptosis and suppressed the proliferation of melanoma cells in vitro, the effects of RIG-I deletion on xenograft tumors in nude mice were investigated. As shown in (Fig. 7A, B), the tumors in mice injected with *RIG-I*-KO cells showed an obviously faster growth rate than those in the controls. The difference in tumor volume was significant 20 days after subcutaneous injection (*P* < 0.01). The proliferative ability of the tumor xenografts was evaluated via histopathological staining for Ki67. The results showed that target deletion of RIG-I could induce proliferation in the tumor xenografts of B16-F10 cells (60.97 ± 13.56 vs. 35.96 ± 7.22%, *P* < 0.001) (Fig. 7C). Subsequently, apoptosis in the tumor tissue was examined using a TUNEL assay. The percentage of apoptotic cells in *RIG-I*-KO tumor xenografts (0.68 ± 0.32%) was significantly decreased compared with that in the control sample (1.61 ± 0.93%) (Fig. 7D). These results demonstrated that RIG-I deletion facilitated tumorigenesis in a xenograft tumor model in vivo.

### RIG-I regulates the growth of melanoma cells partially via MKK/p38 MAPK signaling cascade

**Fig. 8:**
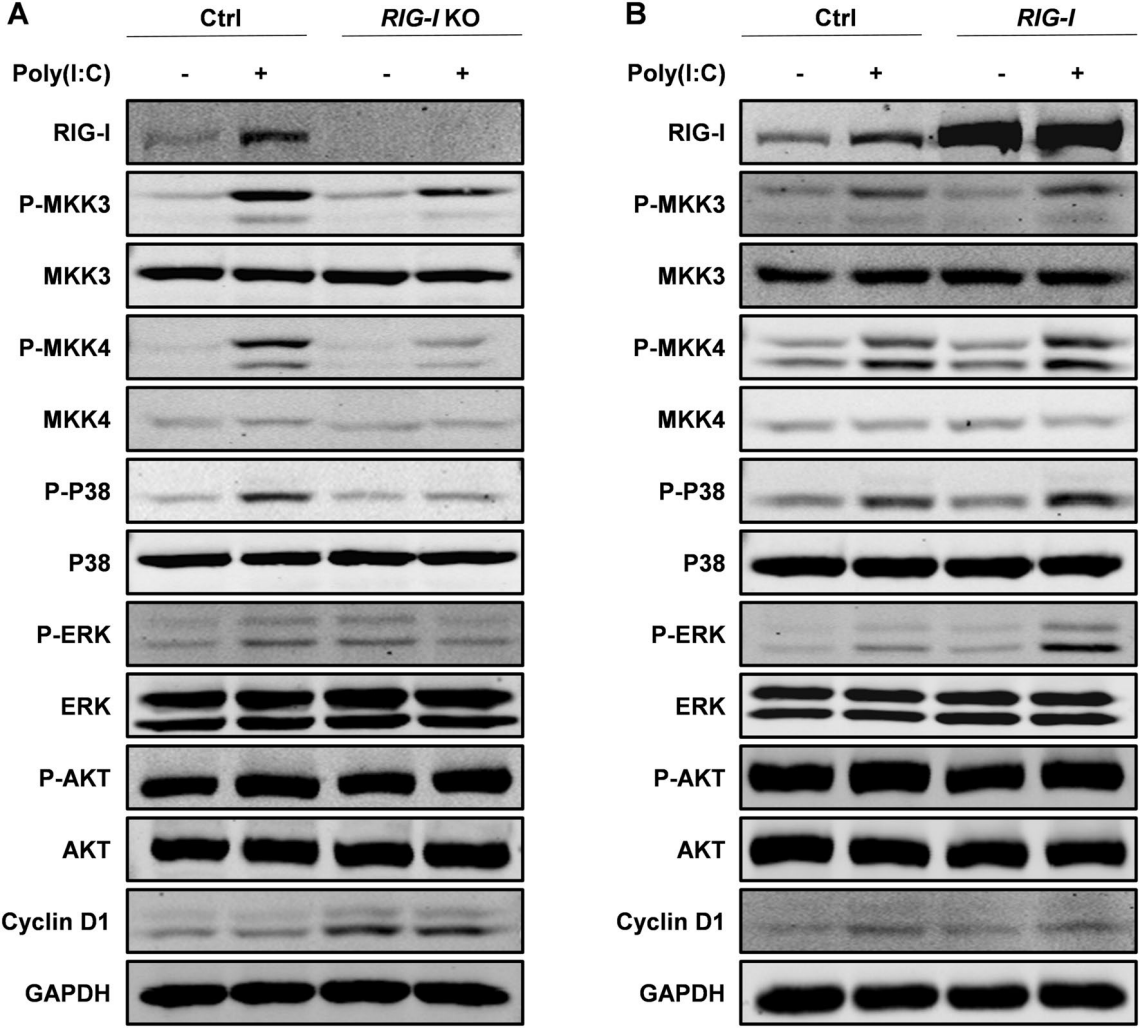
RIG-I exerts antitumor effects through the MKK/p38 MAPK signaling pathway in B16-F10 cells. **A** Ctrl and *RIG-I* KO B16-F10 melanoma cells were transfected with poly I:C (10 μg/ml) or Lipofectamine 3000 for 24 h, respectively. The total and phosphorylated protein levels of MKK3, MKK4, P38, ERK, AKT, and Cyclin D1 were examined by Western blot assay. GAPDH served as a loading control. **B** Ctrl and *RIG-I* overexpression B16-F10 melanoma cells were transfected with poly I:C (10 μg/ml) or Lipofectamine 3000 for 24 h, respectively. The Western blot assay was conducted as described in Panel A. Three independent experiments were performed

To investigate the underlying mechanism through which RIG-I regulates the biological characteristics of melanoma cells, we analyzed the effects of RIG-I on the modulation of the ERK, AKT and p38 MAPK pathways, which have been demonstrated to be critical regulators in the proliferation and apoptosis of cancer cells. The Western blotting results showed that poly (I:C)-induced phosphorylation of p38 MAPK signaling pathway-related proteins, including p38 MAPK, MKK3 and MKK4, was notably decreased following *RIG-I* knockout. Moreover, cyclin D1, a downstream factor of the p38 MAPK signaling pathway that promotes cell cycle progression, was upregulated in *RIG-I*-KO cells (Fig. 8A). Conversely, ectopic overexpression of RIG-I upregulated the poly (I:C)-induced phosphorylation of p38 MAPK, MKK3 and MKK4 (Fig. 8B). These results suggested that the p38 MAPK signaling pathway may be involved in the effects of RIG-I on melanoma.

## Discussion

RIG-I is one of the most common cytosolic pattern recognition receptors, and its functions in the innate immune system have been well documented. However, accumulating evidence suggests that RIG-I is a multifunctional protein that functions far beyond being a pattern recognition receptor and participates in various biological processes, such as cell proliferation, apoptosis, aging, inflammation, detection of bacterial pathogens and even recognition of self mRNAs [16]. In the past several years, there has been increased interest in the potential role of RIG-I in the development and immune therapy of many malignant diseases. Target activation of RIG-I by poly (I: C) or other agonists was previously tested as a therapeutic strategy in some tumors [23, 25]. However, a number of studies have reported that RIG-I has distinct functions in malignant tumors according to their origins [20, 26, 27].

In the present study, the potential role of RIG-I in the biological characteristics of melanoma was assessed using a CRISPR–Cas9-mediated *RIG-I*-KO and a *RIG-I* overexpression B16-F10 cell line. The proliferation, cell cycle, apoptosis, migration and tumorigenic activities in these cells were detected using CCK-8, BrdU incorporation assays, Annexin V/PI staining, Transwell assays and a xenograft tumor model in vivo, respectively. The results indicated that RIG-I suppresses the proliferation but enhances the apoptosis of melanoma cells, both in the spontaneous state and upon poly (I:C)-stimulation. Then these results was confirmed in a *RIG-I* knockdown human melanoma cell line. Furthermore, target deletion of RIG-I results in a faster growth rate of tumor xenografts, and Ki67 staining and TUNEL analysis also confirmed that *RIG-I* knockout promotes melanoma cell proliferation but inhibits melanoma cell apoptosis in vivo.

The MAPK signaling pathway has a central role in the regulation of cell cycling and apoptosis. Poly (I:C) has been proven to activate p38 MAPK through the membrane receptor TLR3 in several cell types. However, the potential role of p38 MAPK in the cytosolic receptor-mediated recognition of poly (I:C) remains to be addressed [28]. To elucidate the detailed molecular mechanism underlying the modulation of melanoma cell growth by RIG-I, we analyzed the expression and phosphorylation levels of p38 MAPK. Western blotting analysis revealed that RIG-I deficiency significantly suppressed the activation of p38 MAPK. We further investigated the expression and phosphorylation of two main MAP kinase kinases upstream of p38 MAPK, MKK3 and MKK4, and found that their activities were inhibited by *RIG-I* knockout. These results were further confirmed in the *RIG-I* overexpression model. Given that p38 MAPK not only negatively regulates cell proliferation but also exhibits proapoptotic function [29, 30], we postulated that p38 MAPK is responsible for the RIG-I-mediated change in cell growth in melanoma cells.

Taken together, the current study presents a novel role for RIG-I in the inhibition of cell proliferation and induction of apoptosis through modulation of the phosphorylation of MKK3, MKK4 and p38 MAPK. Although more precise studies are needed to verify the molecular regulatory mechanism of RIG-I in the pathogenesis of melanoma, to the best of our knowledge, the current study is the first to demonstrate a possible role of RIG-I-mediated activation of the MKK/p38 signaling cascade in the proliferation and apoptosis of melanoma cells. Our findings might deepen the understanding of the antitumor function of RIG-I in melanoma, which could be useful in elucidating the pathogenesis of and developing new therapeutic strategies for this malignant disease.

## Supplementary Information

Below is the link to the electronic supplementary material.

Supplementary file1 (DOCX 989 KB)

## Data Availability

The data used and/or analyzed during the current study are available from the corresponding author on reasonable request.
