# Supplementary material for: RIG-I acts as a tumor suppressor in melanoma via regulating the activation of the MKK/p38MAPK signaling pathway
Source: Hum Cell. 2022 Apr 13;35(4):1071–83. doi: 10.1007/s13577-022-00698-1 (PMC9226095; doi:10.1007/s13577-022-00698-1)
Supplement: Supplementary file 1 — Supplementary file1 (DOCX 989 KB) [file 13577_2022_698_MOESM1_ESM.docx]

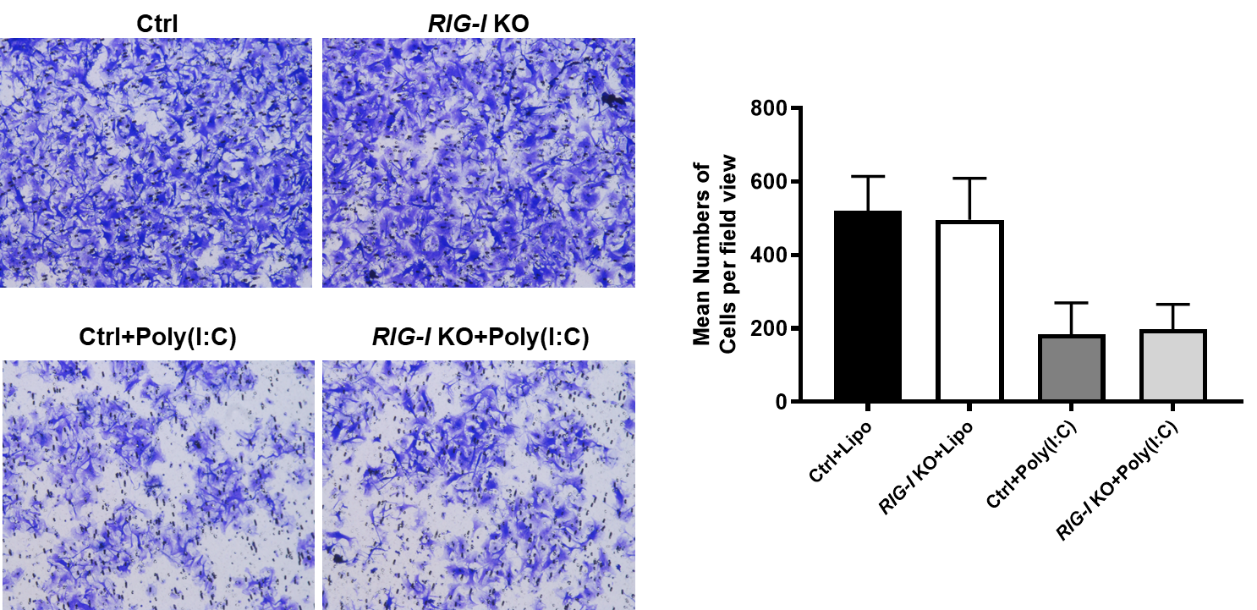


**Fig. S1** *RIG-I* KO has no effects on migration. Ctrl and *RIG-I* KO B16-F10 melanoma cells were transfected with poly I:C (10 μg/ml) or Lipofectamine 3000 for 24 h, then cell migration was evaluated using transwell inserts without Matrigel coating. Representative images and quantitative analysis of the Transwell migration assays. Magnification, x100. Data are presented as the mean ± standard deviation. Three independent experiments were performed.
